# Antibacterial and Anti‐Inflammatory Properties of *Citrus aurantiifolia* Essential Oil–Based Mouthwash: Potential and Cytotoxicity Concerns

**DOI:** 10.1155/sci5/5570684

**Published:** 2026-05-18

**Authors:** Sri Mulyanti, Dewi Sodja Laela, Pramesthi Reitza Navisya Vasall, Nandang Permadi, Dudi Aripin, Amaliya Amaliya, Yaya Rukayadi, Euis Julaeha

**Affiliations:** ^1^ Doctorate Program in Biotechnology, Graduate School, Universitas Padjadjaran, Bandung, 40135, Indonesia, unpad.ac.id; ^2^ Politeknik Kesehatan Kemenkes, Bandung, 40271, Indonesia; ^3^ Research Center for Food Crops, Research Organization for Agriculture and Food, National Research and Innovation Agency, Cibinong, 16911, Indonesia, brin.go.id; ^4^ Department of Conservative Dentistry, Faculty of Dentistry, Universitas Padjadjaran, Jatinangor, 45363, Indonesia, unpad.ac.id; ^5^ Department of Periodontology, Faculty of Dentistry, Universitas Padjadjaran, Jatinangor, 45363, Indonesia, unpad.ac.id; ^6^ Department of Food Science, Faculty of Food Science and Technology, Universiti Putra Malaysia, Selangor, 43400, Malaysia, upm.edu.my; ^7^ Department of Chemistry, Faculty of Mathematics and Natural Sciences, Universitas Padjadjaran, Jatinangor, 45363, Indonesia, unpad.ac.id

**Keywords:** anti-inflammatory effect, antibacterial effect, *C. aurantiifolia* essential oil, cytotoxicity, mouthwash

## Abstract

**Introduction:**

Periodontal disease is one of the most prevalent oral health problems in Indonesia. According to the 2018 National Basic Health Research (RISKESDAS), its prevalence ranges from 73.1% to 75%. The primary cause is dental plaque accumulation, which can be removed chemically using mouthwash. However, most commercial mouthwashes contain synthetic chemicals that may cause adverse effects on oral tissues when used long term. This study aimed to develop a natural‐based mouthwash formulation from the essential oil of lime peel (*Citrus aurantiifolia*), which has been previously reported to possess antibacterial properties. Six formulations were prepared with essential oil concentrations ranging from 1%–20%. Each formulation was tested against *Streptococcus mutans*, *S. sanguinis*, *S. oralis*, *Enterococcus faecalis*, and *Porphyromonas gingivalis*.

**Methods:**

The essential oil was obtained by hydrodistillation and formulated into six mouthwash preparations. The antibacterial activity was assessed using agar disc diffusion for inhibition zones, while MIC and MBC were determined using the broth microdilution method. Physicochemical properties of the mouthwash, including pH, viscosity, and specific gravity, were also analyzed. The most effective formula was further tested in vivo on the gingiva of white rats (*Rattus norvegicus domestica*) to assess its anti‐inflammatory effect based on gingival tissue histology and polymorphonuclear (PMN) cell count. Cytotoxicity was examined using the MTT assay on normal fibroblast cells (3T3‐L).

**Results:**

Formula IV, containing 0.8% essential oil, exhibited the strongest antibacterial activity, particularly against *P. gingivalis*, with a pH of 6.04, specific gravity of 1.0043 g/cm^3^, and viscosity of 1.738 cSt. In vivo tests demonstrated a reduction in gingival inflammation and PMN count, indicating a healing process. The MTT assay confirmed that the formulation was nontoxic to fibroblast cells.

**Conclusion:**

Formula IV represents a safe and effective natural‐based mouthwash for the prevention of dental caries and gingivitis.

## 1. Introduction

Tooth decay and periodontal disease are considered the most prevalent oral diseases. The disease is driven by multifactorial elements, including dental plaque, consumption of high carbohydrates or sugar diets, and poor oral hygiene behaviors [1]. Dental plaque attaches to the tooth surface or any other hard structure in the oral cavity, including calculus, restorations, orthodontic appliances, and implants. It is embedded in a polymer matrix derived from both bacteria and the host, forming a biofilm. The biofilm structure enhances the bacteria’s resistance to the host’s defense system and antimicrobial treatments [2].

Neglected oral hygiene may lead to the persistence of dental plaque. If not removed regularly, the biofilm undergoes maturation, and the resulting pathogenic bacterial complex can lead to dental caries, gingivitis, and periodontitis [3]. Dental caries is a multifactorial disease influenced by biofilm formation and sugar consumption, leading to cyclic demineralization and remineralization of dental hard tissues. Factors such as diet, tooth brushing frequency, saliva, and duration of contact synergistically contribute to acid production. This results in a decrease in pH, which accelerates enamel demineralization, ultimately leading to caries development. Caries can occur throughout life, affecting both primary and permanent teeth, and can damage the tooth crown. On the other hand, gingivitis, an inflammatory response of the gingiva to bacterial plaque accumulation, may occur as a reaction to the same conditions. If left untreated, gingivitis can progress to periodontitis, an irreversible condition that affects the periodontal ligament, cementum, and alveolar bone, further compromising the health of the teeth and supporting tissues. Both conditions are interrelated and require appropriate medical attention to prevent further damage to the teeth and periodontal structures [4–6].

Dental decay and gingivitis are preventable diseases. Removing dental plaque is the key to the prevention of oral disease, which can be performed mechanically by tooth brushing or chemically by using mouthwash containing synthetic active ingredients such as chlorhexidine, cetylpyridinium chloride (CPC), fluoride, and zinc. Nevertheless, long‐term use of synthetic mouthwash may lead to side effects or adverse events on the oral mucous membrane [7, 8]. Therefore, the potential for exploring active compounds with beneficial bioactivity in natural sources warrants further investigation. Active compounds derived from nature have proven to offer significant benefits for oral health. Currently, natural substances that have become active ingredients in oral health products, including garlic, green tea, aloe vera, and cranberries, have been shown to prevent and treat periodontal diseases [9, 10].

Aripin et al. [11] investigated the antibacterial effects of various *Citrus* essential oils, such as *C. limon, C. sinensis, C. aurantiifolia,* and *C. nobilis*, against *Streptococcus mutans*. The results showed that *C. aurantiifolia* exhibited the highest antibacterial activity compared to other *Citrus* species. The antibacterial activity of *C. aurantiifolia* essential oil was supported by components such as d‐limonene, β‐pinene, α‐terpineol, and terpinen‐4‐ol. Another study demonstrated that β‐pinene isolated from *C. aurantiifolia* essential oil also displayed strong antibacterial activity against *S. mutans* [11–13].

In a previous study, the authors developed a mouthwash formulation using essential oil from *C. aurantiifolia* peel as active ingredient at varying concentrations (0.1%–2%). Each concentration was tested against *S. mutans*, and the results showed that the mouthwash formulation with 2% *C. aurantiifolia* essential oil significantly inhibited the growth of *S. mutans* [14]. This study aims to develop mouthwash formulation with variation concentration of *C. aurantiifolia* peel essential oil, against bacteria associated with caries, oral infections, and gingivitis. The bacteria involved include *S. aureus, E. faecalis, S. oralis, S. sanguinis,* and *P. gingivalis.* The parameters evaluated were the inhibition zone against those five bacteria, cytotoxicity of the mouthwash tested on the fibroblast cell line 3T3‐L tissue, and anti‐inflammatory properties tested *in vivo* by observing the number of polymorphonuclear (PMN) cells in gingivitis rats (*Rattus norvegicus domestica*) treated with the mouthwash, as well as its physical properties.

## 2. Materials and Methods

### 2.1. Materials

The peel of *Citrus aurantiifolia* was obtained from Curah Jati Village, Purwoharjo District, Banyuwangi Regency, East Java, Indonesia, during the period of September to October 2023. Ingredients used in the mouthwash formulation—such as Tween 80, peppermint, sodium benzoate, sodium saccharin, and coloring agents—were obtaining from PT Subur Kimia Jaya, Bandung. The target bacteria were cultured at the Central Laboratory of Universitas Padjadjaran, Bandung, Indonesia. Wistar rats of both sexes were obtained from the Inter‐University Center for Education (PAU), Institut Teknologi Bandung (ITB), Bandung. The anti‐inflammatory assay was conducted at the Faculty of Medicine, Universitas Padjadjaran, Bandung. The cytotoxicity test was carried out at the Cell Culture and Cytogenetics Laboratory, Faculty of Medicine, Universitas Padjadjaran, Bandung, Indonesia.

### 2.2. Methods

#### 2.2.1. Preparations of *C. aurantiifolia* Peel Essential Oil

The harvested lime fruits were thoroughly washed, after which the peels were separated and cut into small pieces. The Stahl distillation apparatus was assembled, and the peels of *C. aurantiifolia* were placed into a round‐bottom flask. Water was added until the peels were submerged. Distillation was carried out at 100°C for 3 h in a closed system. Na_2_SO_4_ was added to remove any remaining water [15].

#### 2.2.2. Formulation of Mouthwash

**FIGURE 1:**
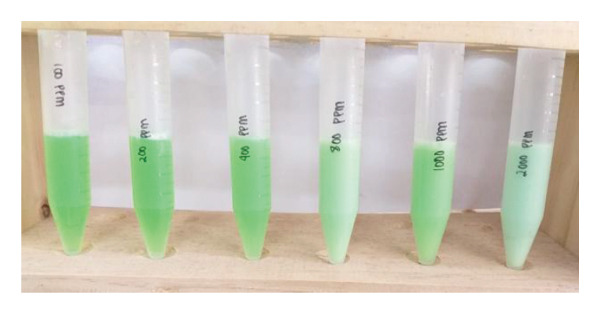
Visualization of mouthwash formula with natural active ingredient essential oil peel of *C. aurantiifolia*.

The mouthwash formulation with *C. aurantiifolia* peel essential oil was conducted following the method described by Mulyanti et al. [14]. The mouth rinse formulations were prepared at concentrations of 0.1%, 0.2%, 0.4%, 0.8%, 1%, and 2% (Table 1 and Figure 1) to determine the optimal activity against S*. mutans, S. aureus, E. faecalis, S. oralis, S. sanguinis,* and *P. gingivalis*.

**TABLE 1:** Formulations of mouthwash supplemented with various concentrations of C. *aurantiifolia* peel essential oil.

Ingredients	Formula					
	I	II	III	IV	V	VI
Essential oil (%)	0.1	0.2	0.4	0.8	1	2
Tween 80 (%)	10	10	10	10	10	10
Peppermint (%)	1	1	1	1	1	1
Na‐benzoate (%)	0.4	0.4	0.4	0.4	0.4	0.4
Na‐saccharin (%)	0.6	0.6	0.6	0.6	0.6	0.6
Coloring (%)	0.2	0.2	0.2	0.2	0.2	0.2
Aquadest (mL)	100	100	100	100	100	100

#### 2.2.3. Physical Property Test

The physical test for mouthwash formula includes pH ranges, type weight, and viscosity of the therapeutic component. The pH meter defines the pH parameter; the picnometer determines the type weight; and the Oswald tube determines the viscosity. Next, viscosity is computed using Oswald’s formula, as follows [13]:

(1) η1η0=t1ρ1t1ρ1,

where *η*_0_ = the viscosity of standard fluid; *η*_1_ = the viscosity of specified fluid; *t*_0_ = the standard fluid flow time; *t*_1_ = the specified fluid flow time; *ρ*_0_ = the standard liquid density; and *ρ*_1_ = the specified liquid density.

#### 2.2.4. Inhibition Zone

The antibacterial activity was evaluated using the disc diffusion method. Mouthwash formulation samples containing C. *aurantiifolia* peel essential oil at concentrations ranging from 0.1% to 2%, chlorhexidine as the positive control, and the mouthwash formulation without essential oil as the negative control were tested against *S. mutans* ATCC 25175, *Staphylococcus aureus* ATCC 6538, *Enterococcus faecalis* ATCC 29212, *Streptococcus oralis* ATCC 6249, *Streptococcus sanguinis* ATCC 10556, and P. gingivalis ATCC 33277. Sterile paper discs (6 mm in diameter) were impregnated with the test formulations and placed onto agar plates previously inoculated with each bacterial strain. The plates were incubated at 37°C for 24 h. Following incubation, the inhibition zones around the discs were measured in millimeters to assess the antibacterial effectiveness of each essential oil concentration.

#### 2.2.5. Minimum Inhibitory Concentration (MIC) and Minimum Bactericidal Concentration (MBC) of *C. aurantiifolia* of Formulation IV

The MIC and MBC tests were conducted using a similar method as described by Rodríguez‐Melcón et al. [16]. In this method, the essential oil from *C. aurantiifolia* peel was tested against bacterial strains including *S. mutans* ATCC 25175, *S. aureus* ATCC 6538, *E*. *faecalis* ATCC 29212, *S. oralis* ATCC 6249, and *S. sanguinis* ATCC 10556. A 96‐well microplate was used, where 100 μL of thioglycolate medium and 100 μL of solvent (dimethyl sulfoxide) were added to each well at a concentration of 100,000 ppm. 2‐fold dilution series were made, and then 100 μL of Formula IV mouthwash was added. Afterward, bacterial suspensions of the aforementioned strains were added to the wells. The microplate was incubated at 37°C for 24 h, and the optical density (OD) was measured using an ELISA reader to determine the MIC based on the percentage of cell inhibition (15%). For the MBC, the test was conducted by subculturing the samples onto Mueller–Hinton agar (MHA) medium and incubating them at 37°C for 48 h. The absence of bacterial growth on the agar plate was considered the MBC.

#### 2.2.6. Cytotoxicity Test

The cytotoxicity test was carried out in the same way as Vajrabhaya and Korsuwannawong [17]. Cytotoxicity was done with the cell line fibroblast 3T3‐L1 that was already cultured. Before performing the MTT assay test, the test materials were removed from each well of the first plate. Then, 50 μL of MTT reagent (5 mg/mL) was added and incubated for 2 h at 37°C in the CO_2_ incubator. The MTT solution was then discarded, and 100 μL of isopropanol was added. The plates were placed on a shaker to solubilize the formations of purple crystal formazan. The absorbance was measured using a microplate reader at a wavelength of 570 nm. The results were used to construct a graph of cell viability percentages against essential oil *C. aurantiifolia* concentrations.

#### 2.2.7. Preliminary In Vivo Anti‐Inflammatory Test

White rats (*R. novergicus domestica)* were randomly allocated into three experimental groups (*n* = 3 rats/group): (i) a negative control group receiving mouthwash base without essential oil, (ii) a positive control group receiving 0.2% chlorhexidine mouthwash, and (iii) a treatment group receiving test formula IV mouthwash. Experimental gingivitis was induced before mouthwash administration by injecting *P. gingivalis* suspension (5 mg in 0.05 mL PBS) into the gingival sulcus at the labial side of the lower incisors using a 30‐G insulin syringe. Each rat received 0.02 mL/day for 5 consecutive days [18]. After gingivitis induction, mouthwash treatments were administered twice daily by pipette at a dose of 2 mL per administration for 7 days. Gingival status was monitored every 2 days through visual inspection for clinical signs of inflammation (e.g., erythema/color change and swelling), as previously described [19].

## 3. Results

### 3.1. Isolation of *C. aurantiifolia* Peel Essential Oil

The isolation of *C. aurantiifolia* from 28.74 kg peel produced 121.2 g of essential oil. The essential oil has a yellow pale appearance and an aroma of orange. The amount of peel *C. aurantiifolia* was determined by the amount required for the other test.

### 3.2. The Formulation of Mouthwash and Physical Test

*C. aurantiifolia* essential oil was utilized as a natural active ingredient with antibacterial properties. The mouthwash formulations were prepared with varying concentrations of *C. aurantiifolia* essential oil, consisting of six formulations at 0.1%, 0.2%, 0.4%, 0.8%, 1%, and 2%, designated as Formulas I, II, III, IV, V, and VI, respectively. All other ingredients remained identical to the mouthwash base (Figure 1). The formulation was developed to identify the concentration with the most effective antibacterial and anti‐inflammatory activity. Among the tested variants, Formula IV (0.8% essential oil) demonstrated the most favorable physicochemical characteristics. It exhibited a pH of 6.04, a specific gravity of 1.0043 g/cm^3^, and a viscosity of 1.7389 cSt, all of which fall within the acceptable range for mouthwash products and indicate a balance between efficacy and user comfort [13, 20].

### 3.3. Antibacterial Activity of Mouthwash Formulas

The effects of the mouthwash formulation were further evaluated against oral pathogenic bacteria associated with dental caries, gingivitis, and oral infections, including *S. aureus, E. faecalis, S. oralis, S. sanguinis,* and *P. gingivalis*. The effect of the mouthwash formula against *S. mutans* has been previously researched by the authors [14]. Antibacterial activity tests were conducted using a positive control with chlorhexidine, a negative control with mouthwash base, and the mouthwash formula containing active ingredient *C. aurantiifolia* peel essential oil (Table 2).

**TABLE 2:** The antibacterial activity of the mouthwash formula with different concentrations of essential oil from *C. aurantiifolia* peel.

Formula	Essential oil (mg/mL)	Zone of inhibition (mm)^∗^					
		*S. aureus*	*E. faecalis*	*S. oralis*	*S. sanguinis*	*P. gingivalis*	S. mutans*^∗∗^*
Control +	1	11.25 ± 0.35^b,d^	10.45 ± 1.06^a,d^	12.8 ± 0.42^c,e^	12.15 ± 0.21^c,d^	23.85 ± 0.42^c,d^	10.1 ± 0.24^a.e^
Control −	0	N.A	N.A	N.A	N.A	N.A	N.A
I	8.3	N.A	N.A	N.A	N.A	N.A	N.A
II	16.68	N.A	N.A	N.A	N.A	8.04 ± 0.30^b,b^	6.15 ± 0.14^a,a^
III	33.36	N.A	N.A	N.A	N.A	9.79 ± 0.30^c,b^	7.08 ± 0.03^b,b^
IV	66.72	8.05 ± 0.71^b,b^	7.4 ± 0.14^a,b^	8.32 ± 0.17^b,b^	7.37 ± 0.17^a,b^	15.95 ± 0.54^c,c^	8 ± 0.14^b,c^
V	83.4	9.67 ± 0.31^a,c^	9.67 ± 0.31^a,c^	9.72 ± 0.106^a,c^	8.15 ± 0.21^a,c^	17.55 ± 3.65^b,c^	8.3 ± 0.28^a,c^
VI	166.8	11.42 ± 0.31^a,d^	11.42 ± 0.31^a,e^	10.82 ± 0.035^a,d^	10.1 ± 0.707^a,d^	26.88 ± 0.07^b,e^	9.25 ± 0.28^a,d^

*Note:* ^∗^Disc diameter: 6 mm; the first letter of the test is different in the same column; the second is between columns. (+) Control: chlorhexidine. (−) Control: mouthwash without essential oil.

Abbreviation: N.A = not active.

^∗∗^Previous results by Mulyanti et al. [14].

Mouthwash Formulas I–III (containing 1%–4% *C. aurantiifolia* essential oil) exhibited no detectable antibacterial activity against the tested bacterial strains. Antimicrobial activity was first observed in Formula IV (0.08% w/v essential oil), which demonstrated inhibition zones against all bacteria evaluated. The strongest effect was observed against *P. gingivalis*, followed by *S. mutans*, while moderate inhibition was noted for *S. aureus*, *E. faecalis*, *S. oralis*, and *S. sanguinis*. Formulas V and VI, containing higher concentrations of essential oil, showed slightly larger inhibition zones but did not markedly outperform Formula IV.

### 3.4. MIC and MBC of *C. aurantiifolia* Peel Essential Oil

Table 3 shows that the MIC values against the test bacteria ranged from 1.3 to 5.2 mg/mL; mouthwash Formula IV was highly sensitive to *S. oralis* (1.3 mg/mL), followed by *S. mutans* (2.6 mg/mL), and other bacteria (5.2 mg/mL). The MBC of mouthwash Formula IV ranged from 5.2 to 10.4 mg/mL. It was more effective against *S. mutans, E. faecalis, S. oralis,* and *S. aureus* (5.2 mg/mL), as well as *S. sanguinis* (10.4 mg/mL). However, it was difficult to observe the effects on *P. gingivalis* because MHA does not support its growth. This bacterium requires enriched media, such as blood agar supplemented with trypticase soy agar, to develop optimally [21].

**TABLE 3:** The MIC and MBC values of *C. aurantiifolia* peel essential oil.

No.	Bacteria	MIC (mg/mL)	MBC (mg/mL)
1.	*Streptococcus mutans*	2.6	10.4
2.	*Staphylococcus aureus*	5.2	5.2
3.	*Enterococcus faecalis*	5.2	10.4
4.	*Streptococcus oralis*	1.3	5.2
5.	*Streptococcus sanguinis*	5.2	10.4
6.	*Porphyromonas gingivalis*	N.d	N.d

Abbreviation: N.d, not detected.

### 3.5. Anti‐Inflammatory Activity of Mouthwash Formula IV

**FIGURE 2:**
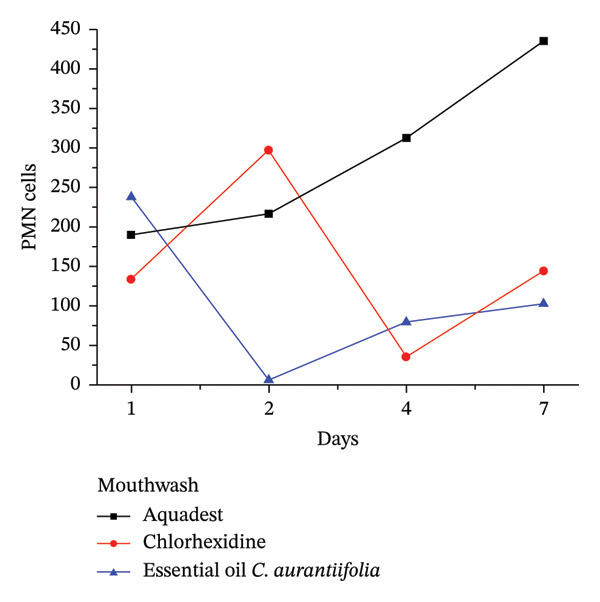
Number of PMN cells after being treated with formula IV, chlorhexidine, and water. As seen in Figure 3, the test rat gums were visibly inflamed.

**FIGURE 3:**
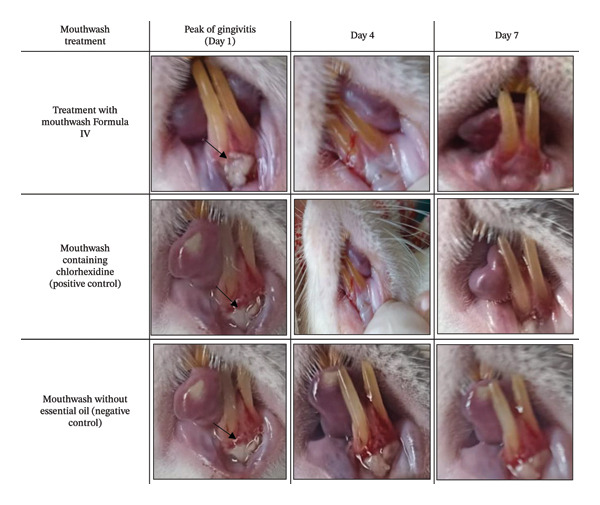
The appearance of inflammation in *R. norvegicus domestica* after receiving *P. gingivalis* injections and the influence of assigning as negative control, positive control, and Formula IV on its inflammation levels.

The anti‐inflammatory effect of Formula IV was evaluated *in vivo*using a gingivitis rat model induced by *P. gingivalis*. As shown in Figure 2, treatment with Formula IV resulted in a reduction of gingival inflammation compared with the negative control (mouthwash base without essential oil). The anti‐inflammatory response observed with Formula IV was comparable to that of the positive control (0.2% chlorhexidine). Histological analysis confirmed a marked decrease in PMN cell infiltration (Figure 3). Formula IV demonstrated an earlier and stronger suppression of PMN accumulation than chlorhexidine, indicating superior anti‐inflammatory potential. These findings suggest that *C. aurantiifolia* essential oil at 0.8% is effective in modulating inflammatory responses associated with gingivitis.

*In vivo* observation (Figure 3) revealed that Formula IV markedly reduced gingival inflammation by day 7, accompanied by clear signs of gingival recovery. By comparison, chlorhexidine treatment produced only partial improvement, while inflammation persisted in the negative control group.

### 3.6. Cytotoxicity

**FIGURE 4:**
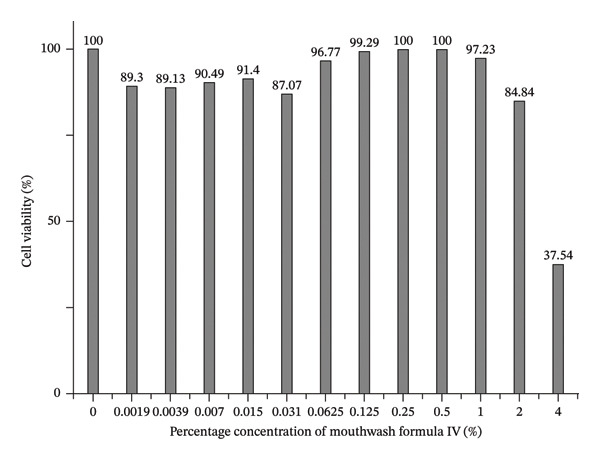
Effects of Formula IV treatment in various concentrations (%) on the viability of normal 3T3‐L fibroblast cells.

Formula IV (0.8% *C. aurantiifolia* essential oil) was tested using a two‐fold serial dilution method (Figure 4) to evaluate its cytotoxicity against 3T3‐L fibroblast cells. The tested concentrations ranged from 0.4% to 0.0019% (w/v) essential oil. As shown in Figure 5, fibroblast viability remained above 90% at concentrations below 0.1%, indicating that the mouthwash is safe at these levels. However, cell viability decreased to 84.08% at 0.2% and further declined to 37.5% at 0.4%, demonstrating a dose‐dependent cytotoxic effect.

FIGURE 5Morphology of normal 3T3‐L fibroblast cells after exposure to mouthwash Formula IV containing *C. aurantiifolia* essential oil at concentrations of 0.0625%–0.4%.

**Figure:**
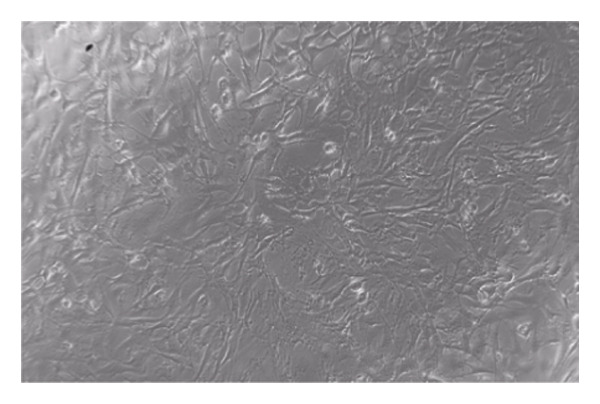
(a)

**Figure:**
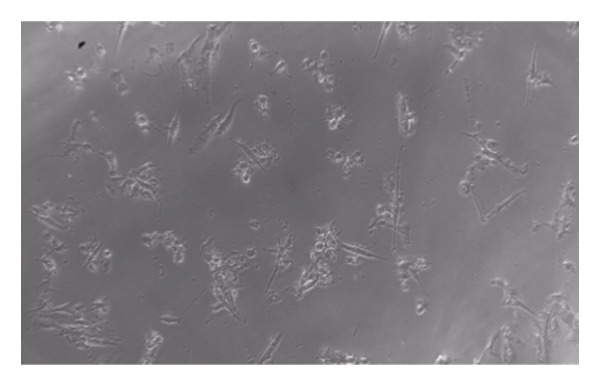
(b)

## 4. Discussion

Mouthwash is one of the most widely used methods for maintaining oral and dental health due to its practical application. Currently, natural antibacterial agents as active ingredients are increasingly favored over synthetic compounds, as natural compounds generally produce fewer side effects even with long‐term use. The essential oil of *C. aurantiifolia* peel has been reported to possess antibacterial activity supported by its chemical constituents, such as d‐limonene and β‐pinene [10, 22]. According to Permadi et al. [12], the antibacterial activity of *C. aurantiifolia* peel essential oil is not solely attributed to d‐limonene but is also influenced by synergistic interactions among its constituents. Based on these considerations, the present study utilized an essential oil containing a mixture of constituents, rather than a single purified active compound, to enhance antibacterial efficacy while minimizing cytotoxicity, aiming to develop a safe and effective mouthwash ingredient.

The essential oil extracted from *C. aurantiifolia* peel by using hydrodistillation in this study yielded 0.86%, which falls within the acceptable range of 0.3%–0.9% [23]. A total of six mouthwash formulations containing *C. aurantiifolia* essential oil were prepared and evaluated for various medicinal properties. Based on the antibacterial activity tests presented in Table 2 against oral pathogenic bacteria, mouthwash Formulas IV (0.8% essential oil), V (1.0%), and VI (2.0%) exhibited strong antibacterial activity against all tested bacterial strains. Among them, Formula IV was selected as the formulation of interest, as higher concentration of *C. aurantiifolia* peel essential oil in Formulas V and VI may disrupt the balance of the oral microbiota, which plays critical role in preventing colonization by pathogenic species [22]. The antibacterial activity of essential oils is generally attributed to their ability to damage bacterial cell walls, thereby altering membrane proteins and the cytoplasmic membrane [24]. According Yan et al. [25], this mechanism is associated with low‐molecular‐weight and highly lipophilic compounds present in essential oils, which can readily penetrate bacterial membranes and compromise cell integrity. Another possible mechanism involves interference with bacterial defense enzymes as well as enzymes essential for cellular growth and development [26, 27].

Among the constituents of *C. aurantiifolia* essential oil, d‐limonene, one of its major components, plays a central role in antibacterial activity. Limonene acts by disrupting bacterial cell membranes and reducing the activity of key enzymes such as Na^+^‐K^+^‐ATPase and Ca^2+^‐Mg^2+^‐ATPase, which are essential for maintaining ion balance, cellular respiration, and energy synthesis [24, 28]. As a result, intracellular ATP levels decline, impairing energy metabolism. Simultaneously, limonene increases membrane permeability, causing the leakage of ions and essential metabolites. The combined effects of ATP imbalance, enzyme inhibition, and membrane disruption compromise bacterial viability, inhibit growth and reproduction, and ultimately lead to cell death. This compound exerts both bacteriostatic effects, which inhibit bacterial proliferation and allow the host immune system to clear pathogens, and bactericidal effects, which directly kill bacterial cells [12, 26, 27]. This dual mechanism is consistent with the MIC values (1.3–5.2 mg/mL) and MBC values (5.2–10.4 mg/mL) obtained in this study, confirming the strong antibacterial potential of Formula IV. Furthermore, the presence of peppermint oil provides complementary antibacterial activity, thereby enhancing the overall efficacy and safety of the formulation as a natural mouthwash [10].

The physicochemical evaluation of Formula IV mouthwash included measurements of pH, density, and viscosity to assess its safety and suitability for oral application [13]. Formula IV exhibited a pH of 6.04, which is within the natural oral pH range (5.5–7.9) and close to the recommended range for herbal formulations [5, 6], thereby minimizing the risk of mucosal irritation [29]. The density (1.002 g/cm^3^) and viscosity (0.912 cSt) values of Formula IV were close to those of water, ensuring comfort, ease of rinsing, and uniform distribution within the oral cavity. These characteristics confirm that the formulation fulfills essential criteria related to safety, user acceptability, and effectiveness as a mouthwash preparation [13, 30].

Subsequently, to evaluate the safety of the mouthwash, an in vivo study was conducted using *R. norvegicus* as the experimental model. The animals, induced by injection of *P. gingivalis* bacteria, developed inflammation that progressed into gingivitis. Gingivitis is an inflammatory condition of the gums caused by plaque accumulation in the gingival sulcus and adjacent areas. If left untreated, the infection can advance into a more severe inflammatory state, characterized by periodontal pocket formation, loss of gingival attachment, and alveolar bone degeneration [31]. The inflammatory process occurs when *P. gingivalis* penetrates the gingival sulcus tissue or surrounding areas, thereby activating proinflammatory components of PMN cells such as neutrophils, eosinophils, and basophils [6, 18, 32].

Inflammation is the body’s response to pathogens and environmental stress, crucial for regulating wound healing. Gingivitis, an inflammation of the gums, is caused by plaque accumulation in the gingival sulcus and adjacent areas. Untreated infections can lead to severe inflammatory conditions characterized by periodontal pocket formation, loss of gingival attachment, and alveolar bone degeneration [33]. The inflammatory process occurs when *P. gingivalis* bacteria penetrate the gingival sulcus tissue or surrounding areas, activating proinflammatory components of PMN cells like neutrophils, eosinophils, and basophils [6, 31, 32].

The anti‐inflammatory effect of mouthwash Formula IV was evaluated in comparison with chlorhexidine and a mouthwash without *C. aurantiifolia* peel essential oil (negative control) in the management of gingivitis. The results indicated that chlorhexidine initially suppressed PMN cell accumulation; however, PMN levels increased again on the following day. Formula IV also reduced PMN infiltration during the observation period and showed an early response by day 2. Although PMN levels increased at later time points, the overall trend remained comparable to that observed with chlorhexidine. These findings suggest that Formula IV exhibits promising anti‐inflammatory activity and may contribute to the reduction of gingival inflammation. In contrast, the negative control did not show a reduction in PMN levels (Figure 2). The changes in PMN infiltration under each treatment corresponded with the gingival inflammatory condition observed in the mice (Figure 3). These findings are consistent with a previous report by Sreenivasan and Haraszthy [30], which indicated that reducing PMN infiltration lowers the risk of chronic inflammation, and further suggest a synergistic effect with the antibacterial activity of the formulation against gingivitis‐associated pathogens.

The cytotoxicity assay was performed to evaluate the safety of the mouthwash formulation prior to potential clinical application. Formula IV, which previously demonstrated the strongest antibacterial activity, was selected for cytotoxicity testing against normal 3T3‐L fibroblast cells to assess its safety profile. The results showed that cell viability remained high at lower concentrations, particularly within the range of 0.0625%–0.1%, where viability ranged from 96% to 100%, indicating a relatively safe concentration range. In contrast, a marked decline in cell viability was observed at higher concentrations, with viability decreasing to 37.5% at 0.4%. According to the commonly accepted criterion, cell viability below 50% is considered indicative of cytotoxicity. These findings indicate that higher concentrations of the formulation may induce cytotoxic effects under prolonged exposure conditions. However, the cytotoxicity assay involved continuous exposure of the cells to the formulation for 24 h, whereas in actual use, mouthwash is typically retained in the oral cavity for only 30 s to 1 min before being expelled. As noted by Sazonova et al. [34], such short exposure times do not cause significant toxic effects, thereby reinforcing the conclusion that Formula IV is safe for use as a mouthwash under normal application conditions. Therefore, the cytotoxic effects observed under the *in vitro* assay conditions should be interpreted with caution and may not directly reflect the biological response during routine oral application. Under practical conditions, the much shorter contact time is expected to reduce the likelihood of significant cytotoxic effect. Furthermore, clinical studies are warranted to validate its long‐term efficacy and safety in human use [35].

## 5. Conclusion

Among six mouthwash formulations containing *C. aurantiifolia* peel essential oil, Formula IV (0.8%) exhibited the strongest antibacterial activity against oral pathogens, including *S. mutans, S. sanguinis, S. oralis, S. aureus, E. faecalis*, and *P. gingivalis*, and also demonstrated anti‐inflammatory effects. Its physicochemical properties met the requirements for a safe and effective mouthwash, while *in vivo* and cytotoxicity assays confirmed its biocompatibility within the concentration range of 0.0625%–1%. These findings suggest that Formula IV is a promising natural‐based mouthwash for preventing dental caries and gingivitis.

## Funding

The authors declare that no external funding was received for this study.

## Conflicts of Interest

The authors declare no conflicts of interest.

## Data Availability

The data that support the findings of this study are available from the corresponding author upon reasonable request.
